# Assessment of the kinesiotherapy’s efficacy in male athletes with calcaneal apophysitis

**DOI:** 10.1186/s13018-017-0637-5

**Published:** 2017-10-06

**Authors:** Ersin Kuyucu, Barış Gülenç, Hüseyin Biçer, Mehmet Erdil

**Affiliations:** 1 Orthopaedics and Traumatology Department, Medipol University, Tem Avrupa Göztepe çıkışı/Bağcılar, Istanbul, Turkey; 2 Physiotherapy Department, Medipol University, Tem Avrupa Göztepe çıkışı/Bağcılar, Istanbul, Turkey

## Abstract

**Background:**

The aim of the present study was to assess the efficacy of kinesiotherapy used for treating various disorders in athletes on pain and pedal functions in patients with calcaneal apophysitis.

**Methods:**

This prospective randomized controlled study included 22 patients with calcaneal apophysitis aged 8 to 16 years presenting with heel pain among junior athletes of a professional football club. The patients were randomly grouped into two groups, with one group receiving sham tape only and the other kinesio tape. American Orthopedic Foot & Ankle Society (AOFAS) and visual analog scale (VAS) scores were recorded before and after the treatment.

**Results:**

The preoperative VAS score of the kinesio tape was 7, and AOFAS score was 62.4; the corresponding figures of the sham group were 6.81 and 70.5, respectively. The kinesio-tape group had a better AOFAS scores at 1st and 3rd month (*p* < 0.05). Posttreatment AOFAS score was 99.7 ± 0.9 for the kinesio-tape group and 97.4 ± 3.9 for the sham-tape group. Posttreatment VAS score was 0.1 ± 0.3 for the kinesio-tape group and 0.4 ± 0.5 for the sham-tape group (*p* > 0.05).

**Discussion:**

Conservative treatment modalities are preferentially used for its treatment. Kinesiotherapy is one of the treatment methods for the apophysitis. In the literature, our study is the first prospective randomized trial on the efficacy of kinesio taping in calcaneal apophysitis.

**Conclusions:**

Although kinesio taping can be effectively used for the restoration of ankle functions of athletes with calcaneal apophysitis, its role in pain is limited. Since it lacks serious side effects, it can be used in combination with or as an alternative to pharmacological treatment in this patient group.

## Background

In adolescent athletes, heel pain is a common condition that affects work force [1]. In children belonging to this age group, calcaneal apophysitis is a major cause of heel pain. Calcaneal apophysitis is a self-limiting condition and affects persons of 8–15 years of age who are overweight and physically active. It is rare after the age of 13–15 years when the calcaneal apophysis is ossified [2, 3].

Kinesiotherapy was first invented and developed by Kenzo Kase. Its principle mechanism of action is to regulate regional blood flow and thus eliminating inflammatory cytokines in the region and allowing reparative cells to arrive the region, thereby providing a relief and reducing symptoms. We aimed to study athletes with calcaneal apophysitis to assess the efficacy of kinesiotherapy, which we considered to be a potential alternative to the previously described arch taping approach [4].

## Methods

This prospective study included 22 junior football players who were diagnosed with calcaneal apophysitis with clinical examination and radiography between 2016 and 2017, and all were actively engaged in football playing. Radiologically lateral calcaneal X-rays showed increased sclerosis and fragmentation of calcaneal apophysis. Those with radiologically closed calcaneal apophysis, a body mass index (BMI) greater than 25, comorbidities in addition to apophysitis, and a history of heel trauma or conservative treatment were excluded from the study.

All athletes provided informed consent before study entry. All participants were fully randomized, with one group undergoing kinesio taping and the other form of taping that mimicked kinesio taping but devoid of kinesio taping properties (sham). Both groups were applied stretching exercises, topical analgesic treatment, and massage therapy aimed at heel and plantar fascia. Ages, weight, and height of all patients were recorded.

Pretreatment and posttreatment 1st week and 1st, 3rd, and 6th month visual analog scale (VAS) scores as well as pretreatment, posttreatment 1st, 3rd, and, finally, 6th month American Orthopedic Foot-Ankle Society (AOFAS) scores were recorded in all patients. We used the AOFAS for the ankle and hindfoot. All patients were monitored for recurrences from the onset of treatment to the end of the 6th month. X-rays evaluation was done before taping.

## Tape application

**Fig. 1:**
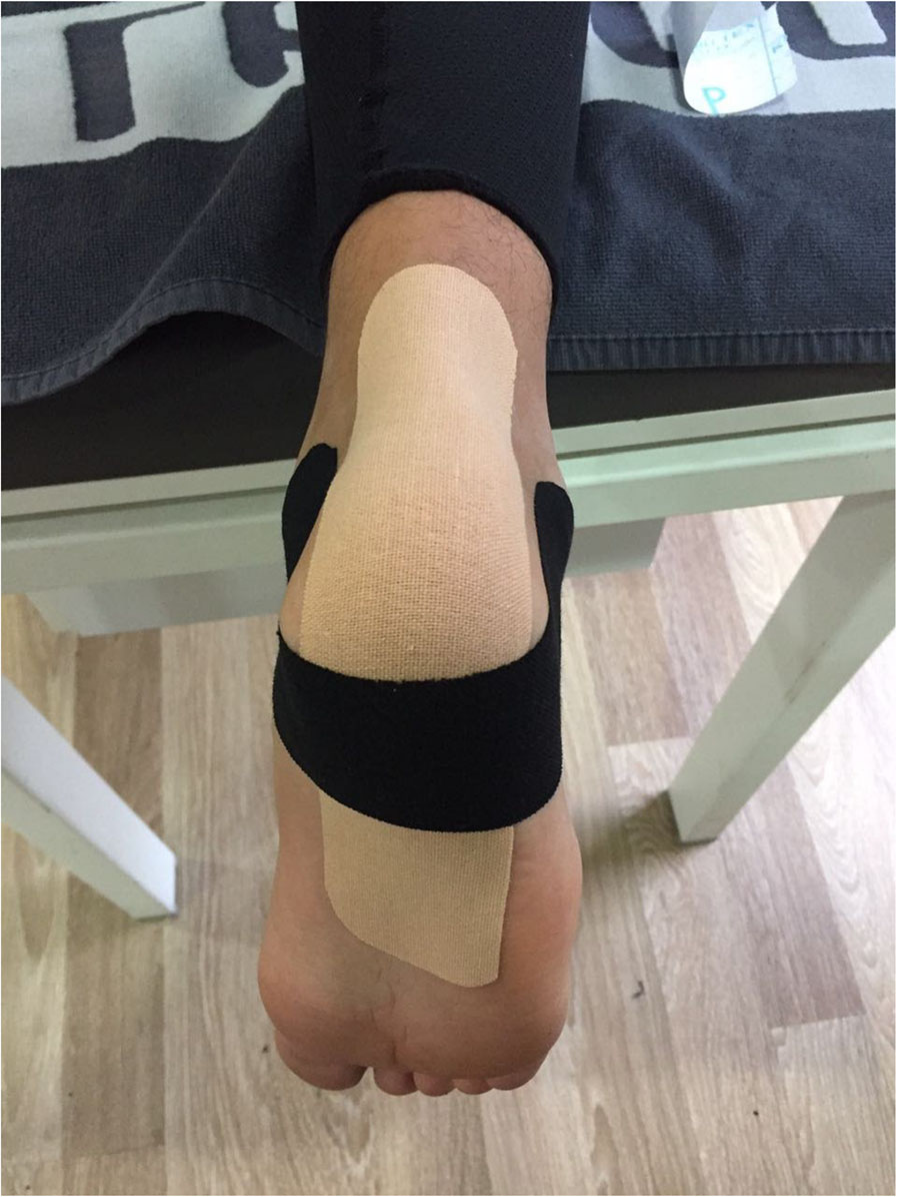
Kinesio taping treatment applied to the Achilles tendon and heel region

Kinesio taping was applied using the mechanical correction method recommended by Kenzo Kase for calcaneal apophysitis; as such, a 4–6-in. tape portion was cut and pulled from both ends to apply moderate to severe tension (50–75%) in a way to intersect the insertion point of the Achilles tendon to the calcaneus perpendicularly. The knee was placed in extension and the ankle in maximum dorsiflexion during the application (Fig. 1) [4].

**Fig. 2:**
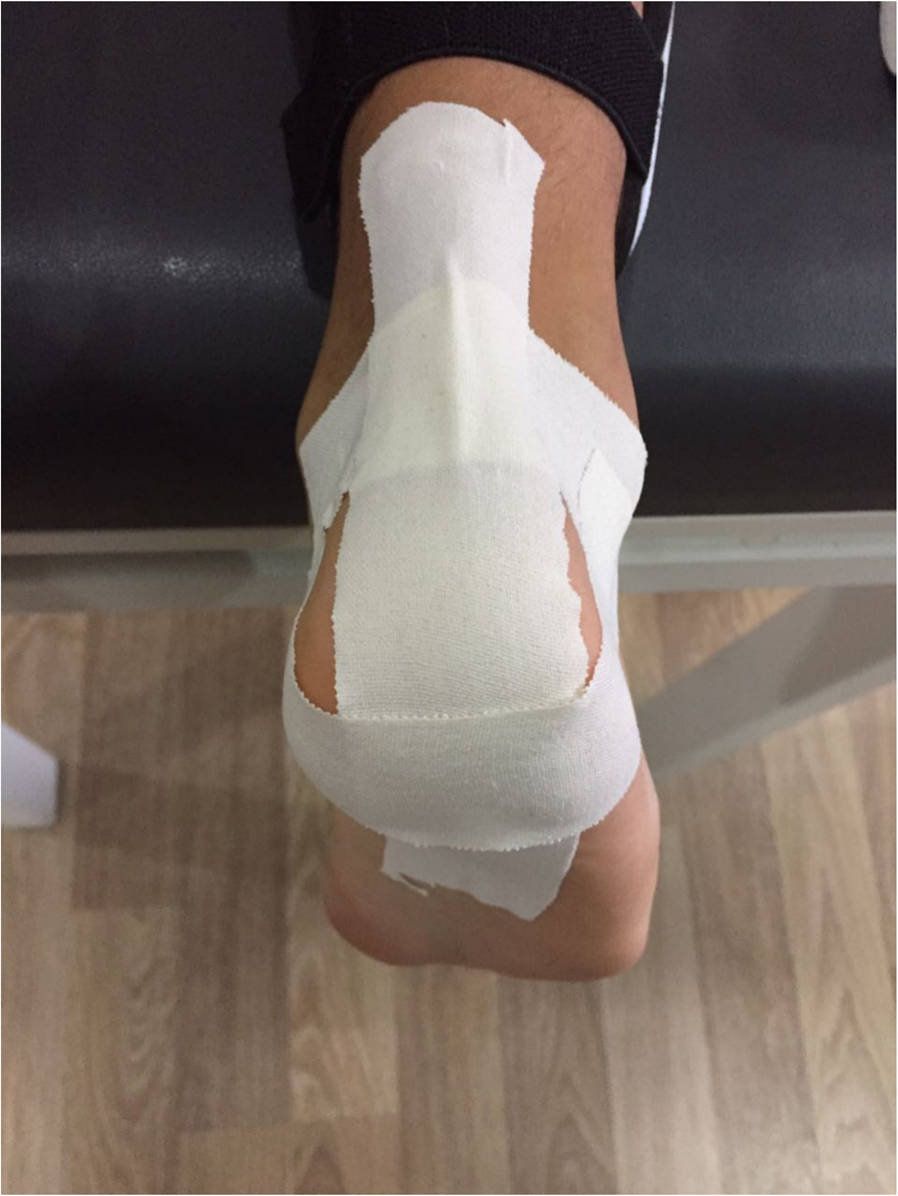
Sham taping applied to the same region

In contrast, sham taping was applied to the same region in a similar manner using Hypafix® (BSN Medical Guilllaime Kroll, Luxembourg) hypoallergenic tape, without applying tension, and with the knee and ankle in extension and dorsiflexion positions as in the kinesio taping (Fig. 2).

Taping was repeated by an expert physiotherapist every 3 days, with 1 day intervals between two consecutive sessions in order to rest the skin. By this way, a total of 12 kinesio taping procedures were carried out and the therapy was terminated.

### Statistical analysis

The descriptive statistics included mean, standard deviation, median, minimum, maximum, frequency, and percentage. The distribution of the study variables was tested using Kolomogorov-Smirnov test. Independent quantitative variables were compared using independent samples *t* test and Mann Whitney *U* test. Dependent quantitative data were analyzed with Wilcoxon test. SPSS 22.0 software package was used for all statistical analyses.

## Results

In this study, we included 27 patients with calcaneal apophysitis with radiologic and clinical diagnosis. Three patients whose BMI > 25 and two patients who have trauma history were excluded. After excluding the above patients, the study was finished with 22 patients. All patients were male. The study population had an age range of 10–16 years, the mean age of the study participants was 13.18 (10–16) years, and their mean BMI was 19.6 (17–22).

**Fig. 3:**
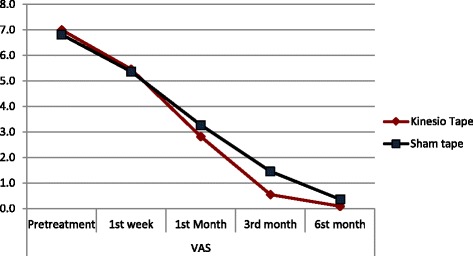
Line graph of VAS alteration during treatment

When we compared the kinesio and sham taping groups, they did not show significant differences in terms of 1st week, 1st month, 3rd month, and 6th month VAS scores (*p* > 0.05) (Table 1). But in itself, Kinesio group showed a significantly decreased 1st week, 1st month, 3rd month, and 6th month VAS scores compared to the pretreatment VAS score (*p* < 0.05). Likewise, in the sham group, the 1st week, 1st month, 3rd month, and 6th month VAS scores showed significant reductions compared to the pretreatment VAS score (*p* < 0.05) (Table 1). Both groups were comparable with respect to the 1st week, 1st month, 3rd month, and 6th month VAS score reductions compared to the pretreatment VAS score (*p* > 0.05) (Table 2, Fig. 3).

**Table 1:** VAS alteration of both groups before and during treatment

	Kinesio tape		Sham tape		*p*
	Median ± SD	Median	Median ± SD	Median	
VAS					
Pretreatment	7.0 ± 0.9	7.0	6.8 ± 1.2	7.0	0.783^a^
1st week	5.5 ± 0.9	6.0	5.4 ± 2.1	6.0	0.893^a^
1st month	2.8 ± 0.6	3.0	3.3 ± 2.0	3.0	0.812^a^
3rd month	0.5 ± 0.5	1.0	1.5 ± 1.2	1.0	0.069^a^
6th month	0.1 ± 0.3	0.0	0.4 ± 0.5	0.0	0.136^a^
Change relative to pretreatment period					
1st week	1.5 ± 0.5	2.0	1.5 ± 1.2	1.0	0.782^a^
Change *p*	0.003^b^		0.011^b^		
1st month	4.2 ± 0.9	4.0	3.5 ± 1.5	4.0	0.199^a^
Change *p*	0.003^b^		0.003^b^		
3rd month	6.5 ± 1.1	6.0	5.4 ± 1.4	5.0	0.057^a^
Change p	0.003^b^		0.003^b^		
6th month	6.9 ± 1.0	7.0	6.5 ± 1.1	7.0	0.341^a^
Change *p*	0.003^b^		0.003^b^		

^a^Mann-Whitney *U* test/*X* ^2^

^2^chi square test

**Table 2:** AOFAS alteration of both groups before and after treatment

	Kinesio tape		Sham tape		*p*
	Median ± SD	Median	Median ± SD	Median	
AOFAS					
Pretreatment	62.4 ± 12.8	58.0	70.5 ± 6.0	71.0	0.176^a^
1st week	83.5 ± 5.5	86.0	77.4 ± 8.9	78.0	*0*.*045* ^a^
3rd month	98.3 ± 3.1	100.0	87.1 ± 8.8	85.0	*0*.*001* ^a^
6th month	99.7 ± 0.9	100.0	97.4 ± 3.9	100.0	0.057^a^
Change relative to pretreatment period					
1st week	21.1 ± 10.7	22.0	6.9 ± 6.5	6.0	*0*.*003* ^a^
Change *p*	0.003^b^		0.006^b^		
3rd month	35.9 ± 13.6	39.0	16.6 ± 9.8	13.0	*0*.*004* ^a^
Change *p*	0.003^b^		0.003^b^		
6th month	37.4 ± 13.1	42.0	26.9 ± 7.7	28.0	*0*.*043* ^a^
Change *p*	0.003^b^		0.003^b^		

^a^Mann-Whitney *U* test/*X* ^*2*^

^2^chi square test

statistical differences of AOFAS scores of both groups (italics)

**Fig. 4:**
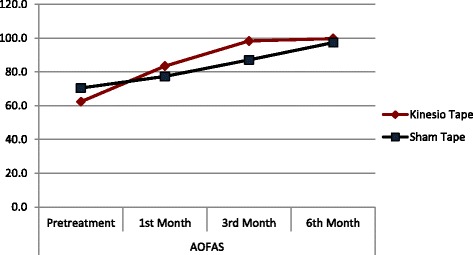
Line graph of AOFAS alteration during treatment

The kinesio and sham groups did not show significant differences between pretreatment and 6th month AOFAS scores (*p* > 0.05). The Sham group showed significant increases in 1st month, 3rd month, and 6th month AOFAS scores compared to the pretreatment AOFAS score (*p* < 0.05) (Table 2). The kinesio group had significantly higher than the 1st month and 3rd month AOFAS scores compared to the sham group (*p* < 0.05) (Table 2, Figs. 3 and 4). Posttreatment AOFAS score was 99.7 ± 0.9 for the kinesio-tape group and 97.4 ± 3.9 for the sham-tape group. Posttreatment VAS score was 0.1 ± 0.3 for the kinesio-tape group and 0.4 ± 0.5 for the sham-tape group (*p* > 0.05).

## Discussion

In this prospective study, we explored no significant differences between both groups with regard to age, weight, height, and body mass index (Table 3). No significant difference was found between the VAS scores of both groups. The results of the present study showed that the AOFAS score used for assessing ankle functions after kinesiotherapy was significantly increased in the kinesiotherapy group compared to the sham group. On the other hand, no significant difference was found between the two groups with respect to score increase.

**Table 3:** Baseline characteristics of the study population with kinesio and sham taping

	Kinesio tape		Sham tape		*p*
	Median ± SD	Median ± SD	Median ± SD	Median	
Age	13.0 ± 2.2	14.0	13.4 ± 2.1	13.0	0.699^a^
Height	168.6 ± 4.9	168.0	173.7 ± 4.3	174.0	0.051^b^
Weight	58.6 ± 3.8	59.0	59.5 ± 3.1	60.0	0.530^b^
BMI	20.6 ± 1.0	20.4	19.8 ± 1.5	19.9	0.131^b^

^a^ *t* test

^b^Mann-Whitney *U* test/*X* ^2^ chi square test

**Fig. 5:**
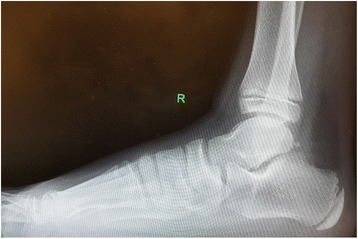
A foot radiogram of a 13-year-old male patient with heel pain. The radiogram shows increased sclerosis of the apophysis of calcaneus

Calcaneal apophysitis pathogenesis is thought to involve inflammation resulting from calcaneal apophysis being subjected to shear forces due to its constant traction by Achilles tendon and plantar fascia, as well as the excessive increase in the intensity of the traction force on apophysis as a result of the Achilles tendon being unable to keep pace with the tibial and fibular growth peak and remaining relatively “short” [5, 6]. The disease is characterized by heel pain, antalgic gait, limited ankle dorsiflexion, and performance loss in young athletes engaged in football and is diagnosed when a patient feels pain in the compression test. Direct films may show increased sclerosis and fragmentation of calcaneal apophysis [3] (Fig. 5). Advanced imaging techniques such as CT, MRI can be used to exclude conditions such as osteomyelitis or malignancy which are included in the differential diagnosis.

Conservative treatment modalities are preferentially used for its treatment. These include non-steroidal anti-inflammatory drugs, massage therapy, cold application, foot orthosis, and arch taping [7].

Direct forces acting on calcaneus apophysis are the traction forces that the plantar fascia and Achilles tendon apply in opposite directions. In normal walking cycle, in the initial contact phase, when the foot contacts the floor, the talonavicular joint displaces distally, the ankle makes internal rotation and pronation, and the plantar fascia becomes tenser. Tense plantar fascia causes a pressure increase in calcaneus apophysis [8–10].

With the continuation of the walking cycle, shortening of the Achilles tendon increases its tension, further increasing load transfer onto calcaneal apophysis [11–13]. The plantar fascia starts in the medial tubercle and attaches to the base of toes. In the late phase of the cycle, when heel elevation takes place, the plantar fascia is folded beneath the convex surface of the metatarsal heads with the dorsiflexion of the metatarsophalangeal joints (the windlass mechanism) [8, 9].

Again, during this phase of walking, the length of the Achilles tendon is significantly shortened and the traction forces on the calcaneus’ center of growth become maximum, causing microavulsions [14, 15].

In our study, we also observed that the improvement of some patients’ complaints occurred too late in the course. The literature data shows an average healing time of several weeks to 2 months [16]. We consider that in persons intensely performing the late swinging phase activity of walking (during shooting or sprinting), such as football players, calcaneal apophysitis is caused by microtraumas in plantar fascia due to repetitive trauma healing with fibrosis, resulting in shortening that slightly further increase the load on calcaneal apophysis. Hence, we think that patients’ complaints are not fully eliminated without this shortening being corrected. What matters here is to determine in which patients fibrosis develops and to predict which athletes would return to sports and when.

Rachel et al. sought to answer the question whether all patients have to undergo radiography to make diagnosis. They reported that pathologies other than calcaneal apophysitis potentially causing severe heel pain were present in 5.1% of 96 patients and that a lateral film should be taken in every patient with heel pain and a positive compression test [17]. We also took lateral foot radiograms for every patient. No athlete was found to have any pathology other than calcaneal apophysitis causing heel pain. Some patients had fragmentation, and some others had increased sclerosis. But we did not classify the X-rays taken for the elimination of other pathologies to exclude or include the patients. Our hypothesis related to this finding is that fragmentation occurs as a result of an excessively increased load on heel apophysis in patients with shortened plantar fascia secondary to sclerosis.

Hundreds of studies have been conducted on the efficacy of kinesio taping since its first introduction; it has been reported to have favorable effects in a considerable portion of these studies [18–25].

We are of the opinion that one of the major reasons of the absence of any significant difference between kinesio taping and sham taping is that the former not altering plantar fascia tension. The force applied by the plantar fascia with the winding wheel mechanism is as effective as the traction force applied by the Achilles tendon on the development of apophysitis [16]. Hence, we suggest that kinesiotherapy on the Achilles tendon alone without taping the plantar fascia would not provide adequate efficacy. Although it appears technically simple to tape the plantar fascia, the efficacy of kinesiotherapy wanes when applied in constantly perspiring feet of athletes with long training hours. We did not apply arch taping or kinesio taping as described in the literature due to difficulties in application.

The major strength of the present study is being the first prospective randomized trial on the efficacy of kinesio taping in calcaneal apophysitis. The others include studying the procedure’s efficacy on professional footballers and assessing not only pain but also providing a functional assessment.

The limitations of the study include a small sample size and the absence of an assessment of arch banding which has been performed in previous studies and has proven efficacy.

## Conclusions

Kinesiotherapy was an effective treatment modality to reduce pain compared to placebo in patients with calcaneal apophysitis. Kinesio taping was significantly more effective than placebo when ankle and foot functional scores were considered.

In addition to taping, treatment modalities such as massage and manual therapy aiming at relieving plantar fascia tension can still be utilized in this age group as the most effective treatment modalities as in the past.

## Abbreviations

AOFAS: American Orthopedic Foot and Ankle Society
BMI: Body mass index
CT: Computed tomography
MRI: Magnetic resonance imaging
VAS: Visual analog scale
